# Interplay between Proline Metabolism and ROS in the Fine Tuning of Root-Meristem Size in *Arabidopsis*

**DOI:** 10.3390/plants11111512

**Published:** 2022-06-05

**Authors:** Sara Bauduin, Martina Latini, Irene Belleggia, Marta Migliore, Marco Biancucci, Roberto Mattioli, Antonio Francioso, Luciana Mosca, Dietmar Funck, Maurizio Trovato

**Affiliations:** 1 Department of Biology and Biotechnology, Sapienza University of Rome, 00185 Rome, Italy; sara.bauduin@gmail.com (S.B.); martina.latini@gmail.com (M.L.); belleggiairene23@gmail.com (I.B.); marta-migliore@libero.it (M.M.); marco.biancucci83@gmail.com (M.B.); roberto.mattioli@uniroma1.it (R.M.); 2 Department of Biochemical Sciences, Sapienza University of Rome, 00185 Rome, Italy; antonio.francioso@uniroma1.it (A.F.); luciana.mosca@uniroma1.it (L.M.); 3 Department of Biology, University of Konstanz, 78457 Konstanz, Germany; dietmar.funck@uni-konstanz.de

**Keywords:** antioxidant enzymes, *Arabidopsis*, hydrogen peroxide, *prodh1 prodh2*, *p5cs1 p5cs2/P5CS2*, proline, reactive oxygen species, root meristem

## Abstract

We previously reported that proline modulates root meristem size in *Arabidopsis* by controlling the ratio between cell division and cell differentiation. Here, we show that proline metabolism affects the levels of superoxide anion (O_2_^•−^) and hydrogen peroxide (H_2_O_2_), which, in turn, modulate root meristem size and root elongation. We found that hydrogen peroxide plays a major role in proline-mediated root elongation, and its effects largely overlap those induced by proline, influencing root meristem size, root elongation, and cell cycle. Though a combination of genetic and pharmacological evidence, we showed that the short-root phenotype of the proline-deficient *p5cs1 p5cs2/P5CS2*, an *Arabidopsis* mutant homozygous for *p5cs1* and heterozygous for *p5cs2*, is caused by H_2_O_2_ accumulation and is fully rescued by an effective H_2_O_2_ scavenger. Furthermore, by studying *Arabidopsis* mutants devoid of ProDH activity, we disclosed the essential role of this enzyme in the modulation of root meristem size as the main enzyme responsible for H_2_O_2_ production during proline degradation. Proline itself, on the contrary, may not be able to directly control the levels of H_2_O_2_, although it seems able to enhance the enzymatic activity of catalase (CAT) and ascorbate peroxidase (APX), the two most effective scavengers of H_2_O_2_ in plant cells. We propose a model in which proline metabolism participates in a delicate antioxidant network to balance H_2_O_2_ formation and degradation and fine-tune root meristem size in *Arabidopsis*.

## 1. Introduction

In addition to its role in protein synthesis, proline is involved in the plant stress response [1] and plant development [2] by accumulating in plant tissues in response to environmental or developmental stimuli, respectively. The accumulation of proline in plant tissues is a highly regulated process relying on the coordinated action of long-distance transport, synthesis in the cytosol, and degradation in the mitochondria. The main synthesis route catalyzes the reduction of glutamate to proline through the sequential action of P5CS and P5CR, while in the mitochondrion, ProDH and P5CDH catalyze the oxidation of proline to P5C and glutamate and the reduction of FAD and NAD^+^ to FADH^2^ and NADH, respectively. The catabolism of proline feeds the electron transport chain, generating ATP through oxidative phosphorylation and superoxide anion (O_2_^•–^) as a by-product of mitochondrial respiration [3,4,5]. Superoxide anions are highly reactive and potentially dangerous ROS molecules, which are rapidly dismutated into hydrogen peroxide, mostly by the action of superoxide dismutase enzymes. In previous work, we showed that proline, among other developmental effects [2], is also involved in the modulation of root meristem size and root growth in *Arabidopsis* [6]. Root growth in plants is controlled by a small number of stem cells surrounding the quiescent center, located in the distal part of the root. These stem cells divide several times in a meristematic zone before they stop their division and start to elongate and differentiate in the elongation/differentiation zone. The boundary between the meristematic zone of cell division and the zone of elongation and differentiation is defined as the transition zone, and its position controls the size of the meristem and consequently the growth rate of the root, which is largely determined by the number of dividing cells [7]. In *Arabidopsis*, the root meristem reaches its final dimension between 5 and 6 days after germination when an equilibrium is reached between cell division in the meristematic zone and cells starting to elongate and differentiate [8,9].

We have shown that *Arabidopsis* mutants defective in proline synthesis, such as *p5cs1-4* [10] or *p5cs1 p5cs2/P5CS2* [11], exhibit roots shorter than wildtype because of a smaller root meristem, and the growth defect is abolished by exogenous supplementation with low proline concentrations ranging from 10 to 20 µM. The effect on meristem size of this amino acid turned out to be uncoupled from the expression of genes controlling cell differentiation at the transition zone, such as *ARR1*, *ARR12*, and *SHY2* [12]. Moreover, the effect of proline on root meristem size was shown to be independent of the action of the plant hormones auxin, cytokinin, and gibberellic acid—the master regulators of root growth in *Arabidopsis*, as shown by pharmacological, molecular, and genetic evidence [9]. On the contrary, proline affects cell division activity in the early stages of postembryonic root development, as revealed by the proline-dependent expression of the G2/M-specific *CYCLINB1;1 (CYCB1;1)* gene [9,11]. Overall, the control of proline on cell division altered the ratio between cell division and cell differentiation, ultimately affecting root elongation.

A similar effect on the root meristem size has been ascribed to some reactive oxygen species (ROS), as reported by Dunand et al. [13] and Tsukagoshi et al. [14] who showed that the ratio between O_2_^•–^ and hydrogen peroxide (H_2_O_2_) affects root meristem growth in a hormone-independent manner, suggesting a possible interaction between proline and ROS in the root elongation process. Moreover, a putative function as ROS scavenger has been long assigned to proline, consistent with the hypothesis that proline and ROS may interact and possibly cooperate to modulate root growth. Indeed, proline has been long proposed as an effective ROS scavenger ever since Smirnoff and Cumbes [15] provided evidence that proline is a powerful scavenger of hydroxyl radicals in vitro, as established by its capability to compete with ascorbate-hydrogen peroxide or xanthine oxidase-hypoxanthine-hydrogen peroxide—two well-known hydroxyl radical scavenging systems. Since this seminal finding, different groups showed that proline could reduce the oxidative damage caused by saline [16], zinc [17], and UV stress [18], as measured by malondialdehyde levels, supporting, but not proving, the idea that proline is important as a non-enzymatic scavenger of free radicals. Later on, Alia et al. [19] showed that proline could reduce singlet oxygen (^1^O_2_)-mediated 2,2,6,6-tetramethylpiperidin oxidation, concluding that proline is an effective ^1^O_2_ quencher, although this claim was confuted by Hamilton and Heckathorn [20].

In addition to a possible direct ROS scavenging, proline could indirectly participate in ROS removal by enhancing the expression and activity of some of the antioxidant enzymes used by plants to maintain ROS levels at optimal levels, such as superoxide dismutases, catalases (CAT), ascorbate peroxidases (APX), glutathione reductase, monodehydroascorbate reductase, dehydroascorbate reductase (DHAR), glutathione peroxidase (GPX), and glutathione S-transferase (GST) [21]. Indeed, several authors have reported a positive correlation between proline and antioxidant enzyme activity [22,23,24]. Overall, the molecular mechanism underlying the putative role of proline as a ROS scavenger is not yet understood, and the question of whether proline exerts a direct or indirect effect on ROS detoxification is still open. In addition to the putative effect of proline on ROS, a reciprocal action of ROS on proline has also been reported [25,26,27]. According to Yang et al. [26], for example, a significant accumulation of proline was found in coleoptiles and radicles of maize seedlings upon exogenous H_2_O_2_ treatment. The increase in proline content was caused either by the upregulation of *P5CS1* and downregulation of *ProDH* at the transcriptional level or by the stimulation of P5CS enzyme activity and inhibition of ProDH enzyme activity at the protein level. Furthermore, Fabro et al. [25] described a hydrogen peroxide-mediated activation of *AtP5CS2* in *Arabidopsis* by avirulent *Pseudomonas* spp interactions. Moreover, Ben Rejeb et al. [27] showed that osmotic and salt stress resulted in both proline and H_2_O_2_ accumulation and, on the basis of the timing of these effects, hypothesized that H_2_O_2_ could act as a secondary messenger upstream of proline biosynthesis.

To shed light on these questions and understand the role of proline as a ROS scavenger and modulator of root growth, we investigated a possible relationship between proline and ROS in the control of the root meristem size.

## 2. Results

### 2.1. Proline Affects the Local Distribution of Superoxide and Hydrogen Peroxide in the Arabidopsis Root

**Figure 1:**
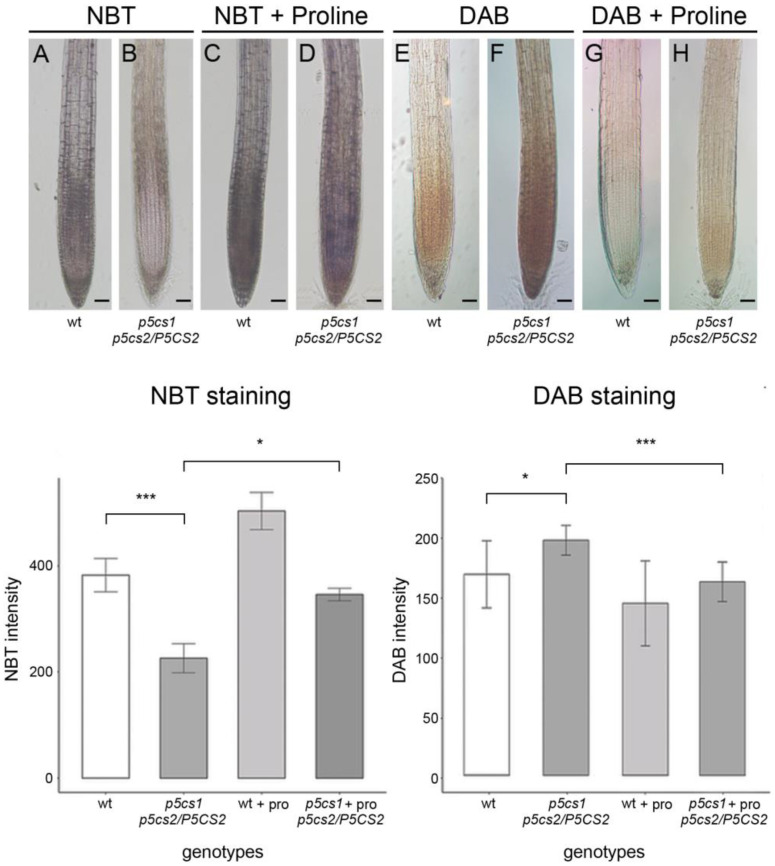
Proline affects the accumulation of superoxide and hydrogen peroxide in the *Arabidopsis* root. Effects of NBT (upper panel, (**A**–**D**), and left side of bottom panel) and DAB (upper panel, (**E**–**H**), and right side of bottom panel) treatment on wildtype and *p5cs1 p5cs2/P5CS2* roots. Wildtype and *p5cs1 p5cs2/P5CS2* roots were treated with 10 µM proline. Bars = 50 µm (**A**–**H**). The staining intensity of the meristematic area of the roots was quantified with ImageJ [28] by scanning digital micrographs acquired at identical illumination and exposure settings. Columns represent the average of ten samples from at least three independent experiments, with a minimum of three technical replicates per experiment. Statistical significance was assessed by Welch Two Sample t-tests, and the *p*-values were corrected for multiple testing using the Bonferroni method. (* *p* < 0.05; *** *p* < 0.001).

To investigate a possible relationship between proline and ROS in the *Arabidopsis* root, we analyzed the *p*-nitrotetrazolium blue (NBT) and 3,3′-diaminobenzidine (DAB) staining levels of superoxide and hydrogen peroxide in *p5cs1 p5cs2/P5CS2* sesquimutant and wildtype roots, with and without exogenous proline supplementation. In wildtype roots treated with NBT, staining was strongest in the meristem and the root cap, whereas staining in the elongation/transition zone and in the quiescent center was weaker (Figure 1A). The short sesquimutant roots [9] exhibited overall weaker coloration, especially in the meristematic region, and the staining was strongest in the elongation/transition zone and the root cap (Figure 1B). DAB treatment, however, indicated opposite effects of P5CS expression on H_2_O_2_. Similar with NBT, wildtype roots showed the strongest coloration in the meristem region, whereas the root cap and the elongation zone showed only weak staining. In sesquimutant roots, the staining was overall stronger and the stained region extended from the meristem into the root cap and the elongation/transition zone (Figure 1E,F, upper panel). Since low levels of exogenous proline have been shown to revert the short root phenotype of *p5cs1 p5cs2/P5CS2* mutants [9,11], we grew wildtype and mutant plantlets on vertical plates containing 10 µM proline to analyze their roots after NBT and DAB staining. As shown in Figure 1D, sesquimutant roots treated with 10 µM proline showed increased NBT staining, with a pattern and intensity similar to untreated wildtype roots. Additionally, wildtype roots showed a trend toward strong NBT staining in response to 10 µM proline, but the difference was not significant (Figure 1C). DAB staining was reduced by proline treatment in wildtype and sesquimutant roots (Figure 1G,H), and again the treated sesquimutant roots resembled untreated wildtype roots (Figure 1E,H).

### 2.2. Proline and Hydrogen Peroxide Have Similar Effects on Root Meristem Size

**Figure 2:**
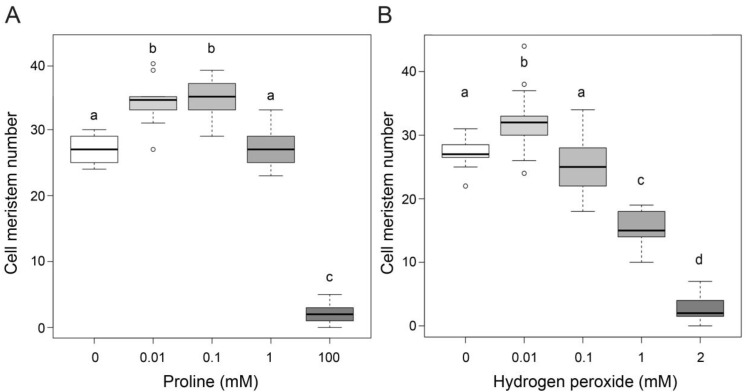
Effects of exogenous proline and hydrogen peroxide on root meristem size. (**A**) Boxplot representation of the average number of cortex cells in each cell file of the root meristem of wildtype *Arabidopsis* treated with an increasing amount of proline. (**B**) Boxplot representing the average number of meristem cells in *Arabidopsis* roots treated with an increasing amount of hydrogen peroxide. Both proline and hydrogen peroxide show similar effects on meristem size, stimulatory at low concentrations, and inhibitory at high concentrations, although, at high concentrations, H_2_O_2_ is more toxic than proline. A one-way ANOVA, followed by a Tukey post-hoc test, confirmed the statistical significance of the effects on root meristem size of either proline or H_2_O_2_. Different letters indicate statistically different group means (*p* < 0.01 between a–b; *p* < 0.001 between a–c, and b–d). Each box represents the mean of at least three independent experiments, each one replicated three times and comprising ten roots.

In our previous work [9,11], we reported that micromolar levels of exogenous proline stimulate root growth and cell division by analyzing root length, the number of cells in the meristem, and GUS activity in roots of CycB1:GUS transgenic *Arabidopsis* plants. The translational fusion between the G2/M-specific cyclin CYCB1;1 and the reporter GUS protein, driven by the CYCB1;1 promoter, shows GUS staining only in actively dividing cells and thereby allows visualization of the meristematic area [29]. In a new set of experiments, we re-analyzed the dose-response curve of root meristem size in a broader range of proline dosages. The curve showed a bell-like shape, with an optimal stimulation of root meristem size occurring at proline concentrations between 10 µM and 100 µM. At 1 mM, no stimulation of meristem size was observed, and at 100 mM, concentrations were inhibitory, leading to a strong reduction in meristem size (Figure 2A) and, consequently, root length (not shown). All the dose-effect experiments were carried out between 5 and 7 DAG when the balance between cell division and cell differentiation is established and the root meristem is easier to analyze. We noticed, that the effects of proline on root meristem size translated into more pronounced effects on root length at longer times (data not shown), suggesting a cumulative long-term effect of proline on root elongation.

A dual influence on plant growth is also exhibited by hydrogen peroxide, which besides its well-known toxic effects at high concentrations [30,31], can stimulate cellular proliferation at low concentrations, as reported by [14,32,33]. To confirm these data and assess whether exogenous hydrogen peroxide could mimic proline effects on root meristem size, we grew wildtype roots in vertical plates supplemented with different H_2_O_2_ concentrations. As shown in Figure 2B, 2 mM, reduced the size of the root meristem to a similar degree as 100 mM proline. At concentrations over 2 mM (not shown) H_2_O_2_ caused an even stronger reduction in meristem size and root length, eventually leading, at concentrations close to 5 mM, to complete inhibition of seed germination. At 10 µM H_2_O_2_, however, we observed a consistent and significant (*p* < 0.001) stimulation of root meristem size and root length with optimal H_2_O_2_ concentrations ranging between 10 and 20 µM (Figure 2B), leading to an increase in cell meristem number from 7 to 18%, depending on the experiments. The stimulatory and inhibitory effects of H_2_O_2_ at low and, respectively high concentrations, resembled those induced by proline, particularly at low concentrations, whereas the threshold concentration for toxicity was lower for H_2_O_2_ than for proline.

**Figure 3:**
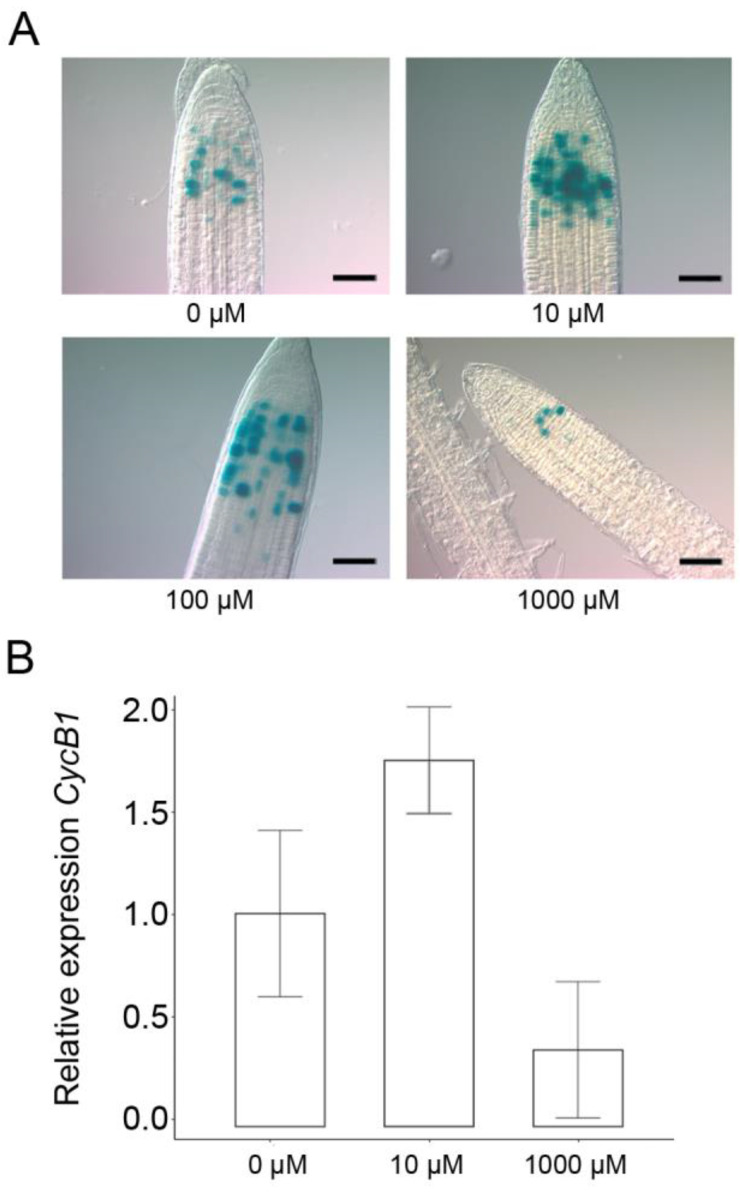
Effects of exogenous hydrogen peroxide on the meristem size of an *Arabidopsis* root. (**A**) Seven-day-old *CYCB1*:GUS plantlets were treated with increasing concentrations of H_2_O_2_ and roots were stained for GUS activity. At 10 and 100 µM H_2_O_2_, the meristem area and the number of dividing cells were increased, whereas, at 1 mM H_2_O_2_, there was inhibition of cell division. H_2_O_2_ concentrations above 10 mM were highly toxic to *Arabidopsis* plantlets, which hardly germinated and grew. Bars = 40 µm. (**B**) RT-qPCR analysis of the expression of *CYCB1;1* in wildtype roots. Transcript levels were normalized to 0 µM H_2_O_2_ and *RCH1*. The expression of the root meristem-specific *ROOT CLAVATA HOMOLOG 1* (*RCH1*) was used as a reference gene to normalize *CYCB1;1* expression over different meristem sizes. Cq-values are the average of three replicates of a representative biological replicate.

Furthermore, with respect to cell division, H_2_O_2_ and proline revealed overlapping effects. Accordingly, micromolar concentrations of exogenous H_2_O_2_ stimulated cell cycle activity, while millimolar additions inhibited it, as indicated by the number of GUS-positive cells in root tips from *CYCB1*:GUS plants (Figure 3A), and by the expression of *CYCB1*;*1*, as analyzed by qRT-PCR (Figure 3B), in wildtype root meristem treated with 10 µM and 1 mM H_2_O_2_. Overall, these results show that H_2_O_2_ has nearly the same effects as proline on root meristem size and root elongation, suggesting that the effects of proline on root meristem size may be dependent on or integrated with hydrogen peroxide.

**Figure 4:**
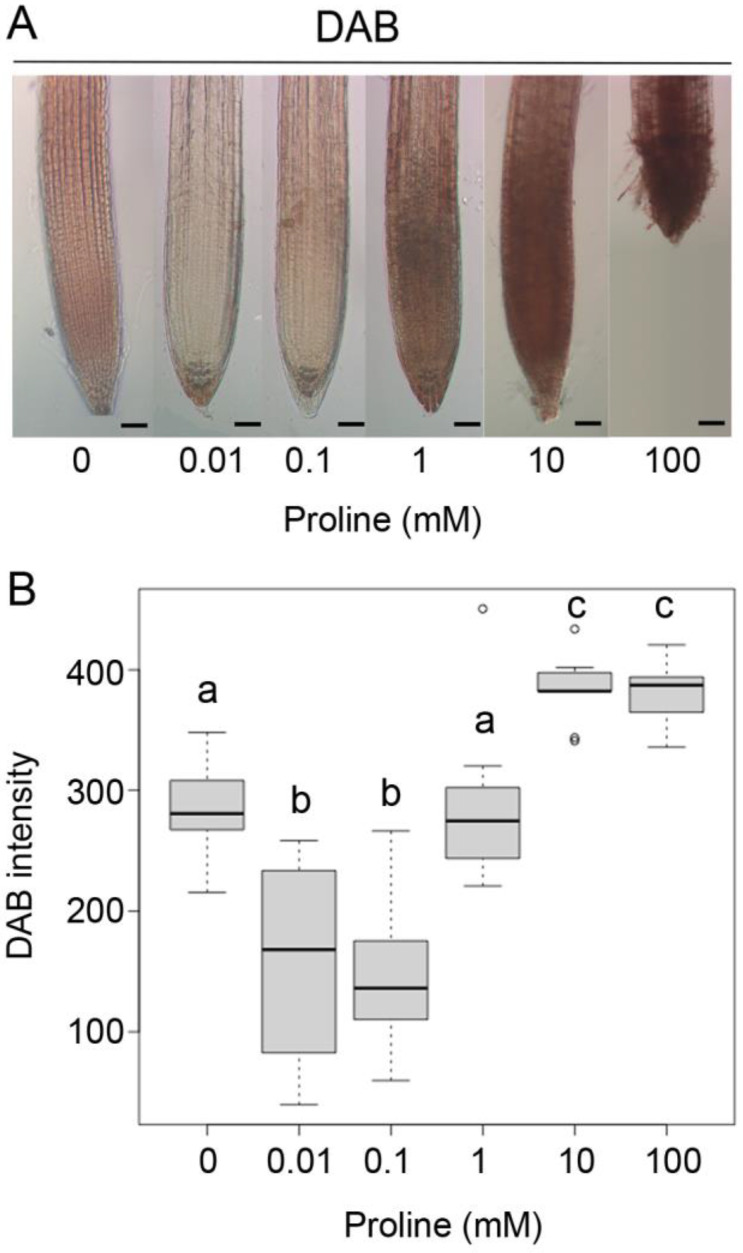
Effect of exogenous proline supplementation on H_2_O_2_ accumulation. (**A**) Wildtype roots were grown for 7 days in vertical plates supplemented with increasing proline concentrations and stained with DAB. Only weak staining is detectable at micromolar proline concentrations, while more intense staining is visible at higher, millimolar concentrations. H_2_O_2_ concentrations above 10 mM, were highly toxic to *Arabidopsis* plantlets, which hardly germinate and grow. Bars: (0–10) = 50 µm; (100) = 20 µM. (**B**) Boxplot representation of the intensity of DAB staining of apical roots from the root cap to the elongation/transition zone. A one-way ANOVA followed by a Tukey post-hoc test identified three groups significantly different (*p* < 0.01 between a–b, and a–c; *p* < 0.001 between b–c). Different letters indicate significant differences among groups. The means represent the average of a minimum of ten samples from three independent experiments replicated at least three times.

To further investigate the correlation between proline and H_2_O_2_, we analyzed the accumulation of H_2_O_2_ in wildtype roots treated with different concentrations of exogenous proline from 10 µM to 100 mM. With 10 and 100 µM exogenous proline, DAB staining was weaker than in untreated roots, indicating the presence of lower levels of H_2_O_2_. At 1 mM proline, the DAB staining intensity was very similar to untreated roots, whereas 10 and 100 mM of exogenous proline caused intense DAB staining, signaling the accumulation of high amounts of H_2_O_2_ (Figure 4).

### 2.3. The Effect of Proline on Root Meristem Size Is Mediated by Hydrogen Peroxide

The correlation between proline and hydrogen peroxide found in the *Arabidopsis* root raises the problem of their epistatic relationships and, in turn, their relative importance in the modulation of root meristem size and root elongation.

**Figure 5:**
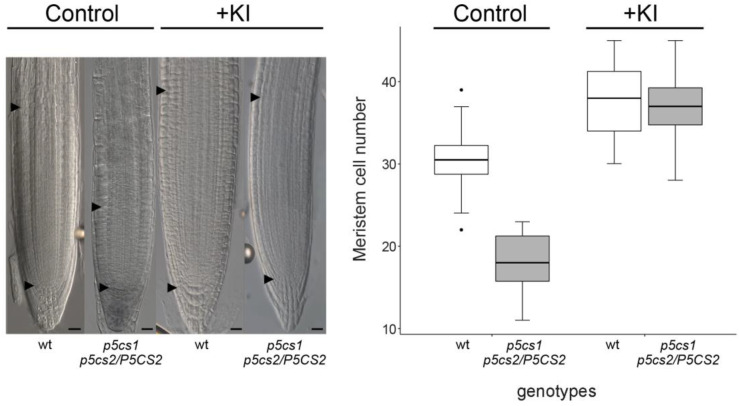
Effect of KI on the root meristem size of wildtype and proline-deficient mutants. (**Left panel**) Root meristem from wildtype and *p5cs1 p5cs2/P5CS2* treated, at 5 DAG, with 10 μM exogenous KI, a strong scavenger of hydrogen peroxide. Black arrowheads indicate the quiescent center (bottom arrowhead) and the transition zone (top arrowhead). Bar = 50 µm. (**Right panel**) Boxplot representation of the average number of meristem cells in a wildtype and *p5cs1 p5cs2/P5CS2* genotype in the presence or absence of KI treatment. A two-way ANOVA analysis revealed a significant interaction between KI and P5CS expression in the modulation of root meristem size. Pairwise comparisons with Tukey post-hoc correction were used to analyze differences between individual samples. All pairwise comparisons were significant at *p* < 0.001, except wildtype plus KI versus *p5cs1 p5cs2/P5CS2* plus KI which was non-significant. Each box represents the mean of at least three independent experiments, each one replicated three times and comprising ten roots.

To test the hypothesis that proline can modulate root meristem size by controlling ROS accumulation in the root apex, and that its effects on root meristem rely on H_2_O_2_ accumulation, we treated *p5cs1 p5cs2/P5CS2* with potassium iodide (KI)—a strong and effective H_2_O_2_ scavenger. The effects of KI on the short-root phenotype of the proline-deficient *p5cs1 p5cs2/P5CS2* mutant were striking (Figure 5). Treatment with 10 µM KI led to an increase in the average number of meristem cortex cells from 30.37 ± 0.78 to 37.25 ± 0.95 in the wildtype and from 18.00 ± 0.4 to 36.79 ± 0.85 in the sesquimutant. Due to a stronger effect of KI on the meristem size of sesquimutant roots, the difference to wildtype roots was no longer present in treated roots. We performed a two-way ANOVA to analyze the combined effect of H_2_O_2_ removal by KI treatment and genotype on root meristem size. The analysis revealed a statistically significant interaction between the effects of KI treatment and those of the genotypes with a different proline content (F value = 97.40, *** *p* = 2^−16^). A simple effect analysis indicated that either KI (*** *p* = 2^−16^) and genotypes (*** *p* = 4.29^−15^) had a significant effect and that KI treatment is the main effector.

**Figure 6:**
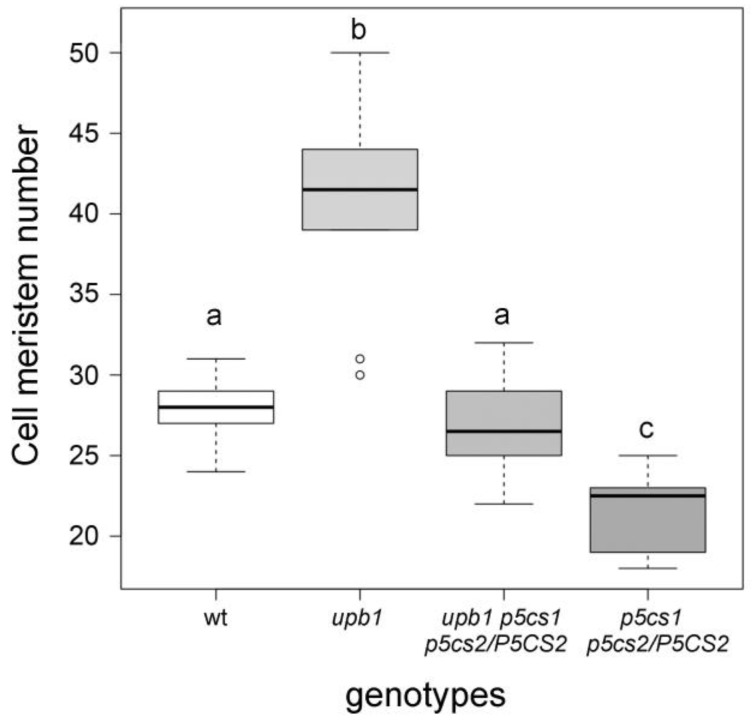
The genetic cross between short-rooted *p5cs1 p5cs2/P5CS2* and long-rooted *upb1* mutant results in roots with meristem sizes of intermediate length. Boxplot representing the average number of cortex cells measured in the root meristem of wildtype, *upb1*, *upb1 p5cs1 p5cs2/P5CS2*, and *p5cs1 p5cs2/P5CS2 Arabidopsis*. A one-way ANOVA followed by a Tukey post-hoc test found a statistically significant increase (*p* < 0.001) in meristem size between the *p5cs1 p5cs2/P5CS2* sesquimutant and the *upb1*, *upb1 p5cs1 p5cs2/P5CS2*, quasi triple mutant. Different letters indicate significant differences among groups. The means represent the average number of meristem cells of ten roots from at least three independent experiments replicated at least three times.

To confirm the interactions between proline and H_2_O_2_ at the genetic level, we crossed *p5cs1 p5cs2/P5CS2* sesqiumutants with *upb1*—a mutant allele of the transcription factor UPBEAT—whose inactivation leads to increased peroxidase activity and, in turn, lower levels of H_2_O_2_ [14], and analyzed the meristem size of the resulting *p5cs1 p5cs2/P5CS2 upb1* quasi-triple mutants. On average, the meristem size of the *p5cs1 p5cs2/P5CS2 upb1* root was very similar to wildtype plants, which was intermediate between *upb1,* and *p5cs1 p5cs2/P5CS2* root meristems, demonstrating that proline synthesis and peroxidase expression exert opposite effects on root meristem size (Figure 6). The most likely interpretation is that the low amount of H_2_O_2_, due to the high peroxidase activity of *upb1* mutants, counteracts the high levels of H_2_O_2_, typical of *p5cs1 p5cs2/P5CS2* mutants, resulting in an intermediate amount of H_2_O_2_ that complements the root elongation defect of the proline mutants.

### 2.4. Role of Proline Catabolism in Proline-Mediated Root Elongation

**Figure 7:**
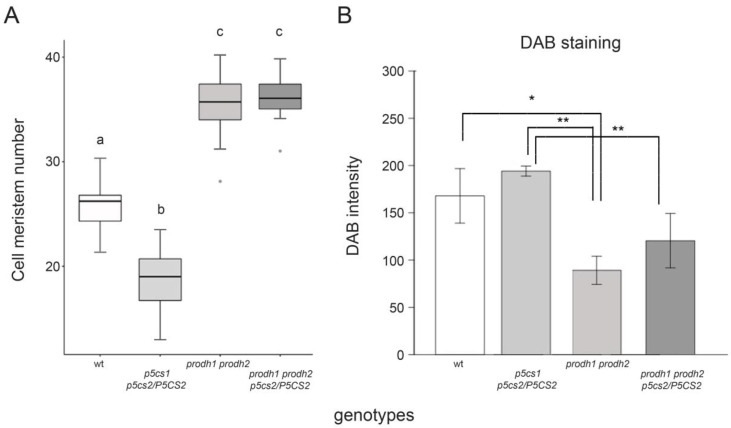
*Arabidopsis* mutants impaired in proline catabolism have large root meristems. (**A**) Boxplot representation of the average number of meristem cells in roots from wildtype, *p5cs1 p5cs2/P5CS2*, *prodh1 prodh2*, and *prodh1 prodh2 p5cs1 p5cs2/P5CS2* plants. Significance among groups was estimated by One-Way ANOVA, followed by a Tukey post-hoc test, which found statistically significant differences among groups a, b, and c (*p* < 0.001). (**B**) DAB staining in wildtype, *p5cs1 p5cs2/P5CS2*, *prodh1 prodh2,* and *prodh1 prodh2 p5cs1 p5cs2/P5CS2* roots. Because variance among groups was not homogeneous, we performed a Welch *t*-test analysis with Bonferroni correction for multiple testing finding significant differences among genotypes (* *p* < 0.05 between *prodh1 prodh2* and wildtype; ** *p* < 0.01 between *prodh1 prodh2* and *p5cs1 p5cs2/P5CS2*, and between and *prodh1 prodh2 p5cs1 p5cs2/P5CS2* and *p5cs1 p5cs2/P5CS2*). Each mean derives from the means of at least three independent experiments, each one replicated three times and comprising ten roots.

The present data show that proline-mediated root elongation is caused by and associated with low levels of H_2_O_2_. The role of O_2_^•–^, however, is less defined in our system. Equally unclear is whether proline itself or its metabolism is involved in the modulation of ROS levels. To clarify these issues, we analyzed the size of root meristems in the double mutant *prodh1 prodh2* [34], in which ProDH activity is almost completely abolished, and thus no proline degradation-dependent production of O_2_^•–^ is expected. We also generated and analyzed the quasi-quadruple mutant *prodh1 prodh2 p5cs1 p5cs2/P5CS2*, with defects in both synthesis and catabolism of proline. The average size of the *prodh1 prodh2* root meristem was significantly larger (35.24 ± 0.84) than the meristem of the wildtype (26.72 ± 1.01; *p* < 0.001) (Figure 7A). A similar phenotype was observed in the quasi-quadruple mutant *prodh1 prodh2 p5cs1 p5cs2/P5CS2*, which exhibited a number of root meristem cells similar to *prodh1 prodh2* (Figure 7A).

To shed light on this unexpected result, we analyzed the root meristems of these mutants by DAB analysis. The average DAB staining of the *prodh1 prodh2* mutant roots (Figure 7B) was very low, indicating significant (* *p* < 0.05) lower levels of H_2_O_2_ in *prodh1 prodh2* mutants compared to wildtype and even less compared to *p5cs1 p5cs2/P5CS2* (** *p* < 0.01), in good correlation with the size of their root meristems. Incidentally, the large root meristem exhibited by *prodh1 prodh2 p5cs1 p5cs2/P5CS2* reinforces the finding that H_2_O_2_ acts downstream of proline and is the main determinant of the effect of proline on root meristem size.

Due to their inability to oxidize proline, *prodh* mutants accumulate this amino acid [35,36], which could be responsible for the large meristem size of the double and quadruple mutants, if proline acted as a direct ROS scavenger to remove inhibitory concentrations of H_2_O_2_ [1]. To assess this possibility, we measured the concentrations of free proline from wildtype and mutant plantlets grown in Petri dishes for seven days.

**Figure 8:**
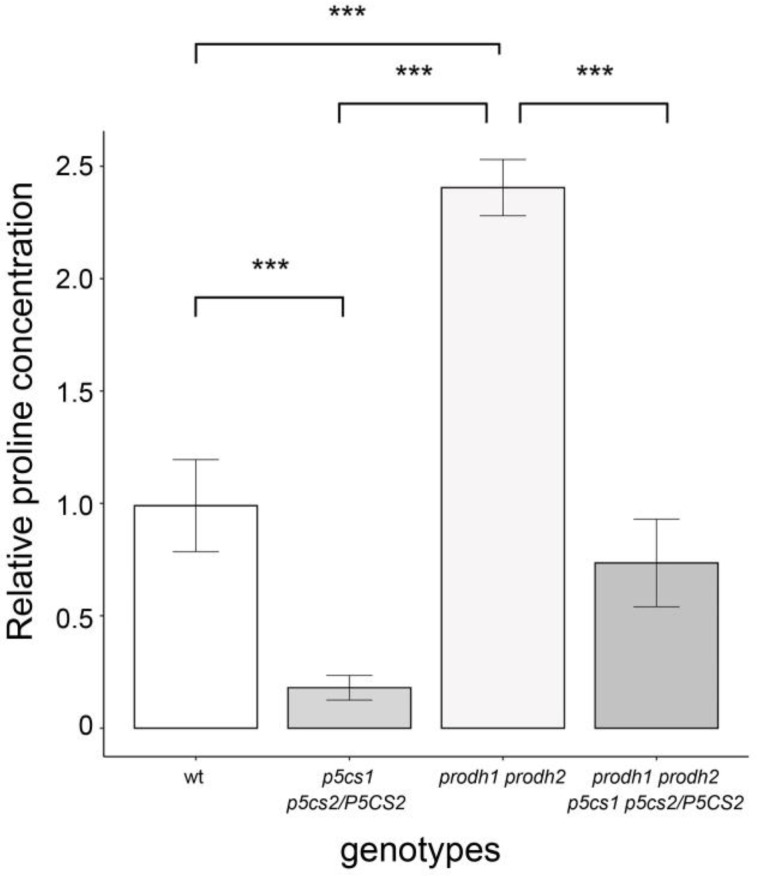
Endogenous proline levels in plantlets from different proline metabolism mutants. Intracellular levels of proline were extracted from 0.5 g (FW) of either wildtype, *p5cs1 p5cs2/P5CS2*, *prodh1 prodh2*, or *prodh1 prodh2 p5cs1 p5cs2/P5CS2* plantlets with 5-sulphosalycidic acid and measured with the Bates assay [37]. The average proline concentrations were derived from three independent experiments, each one with ten samples and three technical replicates. The differences in proline content among genotypes were analyzed with the non-parametric Kruskal–Wallis test, followed by a pairwise Wilcox test which found significant (*** *p* < 0.001) differences among genotypes except for wildtype vs. *p5cs1 p5cs2/P5CS2*, *prodh1 prodh2.* The proline concentrations were normalized to wildtype seedlings.

The results of this analysis are shown in Figure 8. As expected, we found the highest proline content in *prodh1 prodh2* plantlets, with average concentrations two and a half times higher than wildtypes, and the lowest proline content in *p5cs1 p5cs2/P5CS2* plantlets, with average concentrations fivefold less than wildtype. As to the quasi-quadruple mutant *prodh1 prodh2 p5cs1 p5cs2/P5CS*, we found proline levels slightly lower than in wildtype, with average differences not statistically significant.

The relatively low level of proline found in the quadruple mutant, similar if not lower than the wildtype, correlates neither with its large root meristem nor with its low levels of H_2_O_2_, arguing against a direct role of proline as an H_2_O_2_ scavenger. It is interesting to note that although both *p5cs1 p5cs2/P5CS2* and *prodh1 prodh2 p5cs1 p5cs2/P5CS,* are defective in proline metabolism, the former accumulates H_2_O_2_ and has a small root meristem, while the latter has a low H_2_O_2_ content and a large meristem. Since the intracellular proline content of the quadruple mutant is not compatible with a direct role of proline in H_2_O_2_ scavenging, the likely explanation is that in absence of proline catabolism the accumulation of H_2_O_2_ due to mitochondrial respiration is strongly reduced, pointing to the importance of proline catabolism in ROS regulation. Overall, these data confirm the importance of H_2_O_2_ in proline-mediated root elongation and suggest that proline metabolism rather than proline itself is involved in controlling the level of H_2_O_2_ accumulation.

### 2.5. Proline Enhances the Activity of H_2_O_2_-Scavenging Enzymes

**Figure 9:**
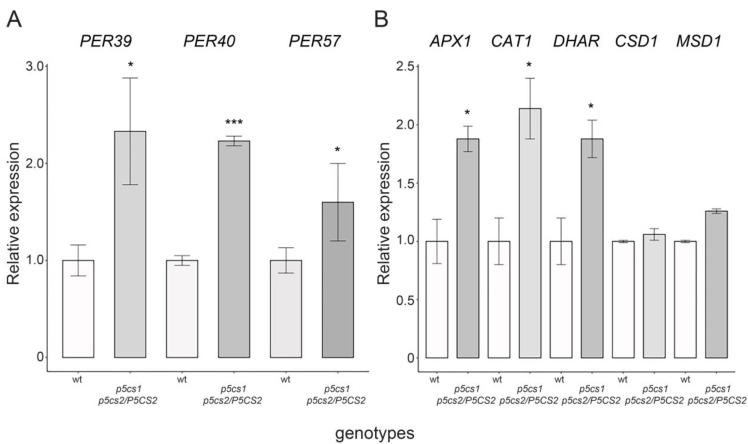
Expression of genes encoding antioxidant enzymes in the root meristem of proline mutants. RT-qPCR was performed on cDNA from apical root portions. The analysis shows upregulation of genes coding for antioxidant enzymes in roots from *p5cs1 p5cs2/P5CS2* mutants relative to wildtype. In (**A**), a significant upregulation of *PER39*, *PER40,* and *PER57* is shown. In (**B**), the genes *APX1*, *CAT1*, and *DHAR* are significantly upregulated. The meristem-specific gene *RCH1* was used as reference control to normalize the RT-qPCR. Error bars indicate Standard Deviation (SD). The Welch Two Sample t-test (wild type vs. mutant lines) was used to assess statistical significance (*** *p* < 0.001; * *p* < 0.05). The data represent the means ± SD of four independent experiments and three technical replicates per experiment.

Two possible mechanisms through which proline might control ROS accumulation rely on either upregulating the genes coding for antioxidant enzymes or enhancing the enzymatic activity of their expression products. To explore the former hypothesis, we compared, through RT-qPCR, sesquimutant and wildtype roots for the expression of genes encoding major antioxidant enzymes such as the root-specific *PEROXIDASE39* (*Per39*), *PEROXIDASE40* (*Per40*), *PEROXIDASE57* (*Per57*), *CATALASE1* (*CAT1*), *ASCORBATE PEROXIDASE1* (*APX1*), *DEHYDROASCORBATE REDUCTASE* (*DHAR*), the cytosolic copper/zinc *SUPEROXIDE DISMUTASE* (*CSD1*), and *MONODEHYDROASCORBATE REDUCTASE1*. Since sesquimutant roots have little proline and accumulate H_2_O_2_, we expected to find the genes coding for antioxidant enzymes expressed at lower levels than wildtypes. On the contrary, the expression of the genes examined in our analysis turned out to be unaffected or upregulated in roots of *p5cs1 p5cs2/P5CS2* sesquimutants compared to wildtype (Figure 9), suggesting that the expression of these genes are either inversely or not correlated to proline. Since most antioxidant genes are known to be induced by H_2_O_2_ [38,39,40], the most likely explanation to account for this result is that the upregulation of the antioxidant genes was caused by the high levels of H_2_O_2_ in the roots of *p5cs1 p5cs2 P5CS2* mutants and we can exclude that the high H_2_O_2_ levels are a consequence of the proline-dependent downregulation of the expression of the analyzed antioxidant enzymes.

**Figure 10:**
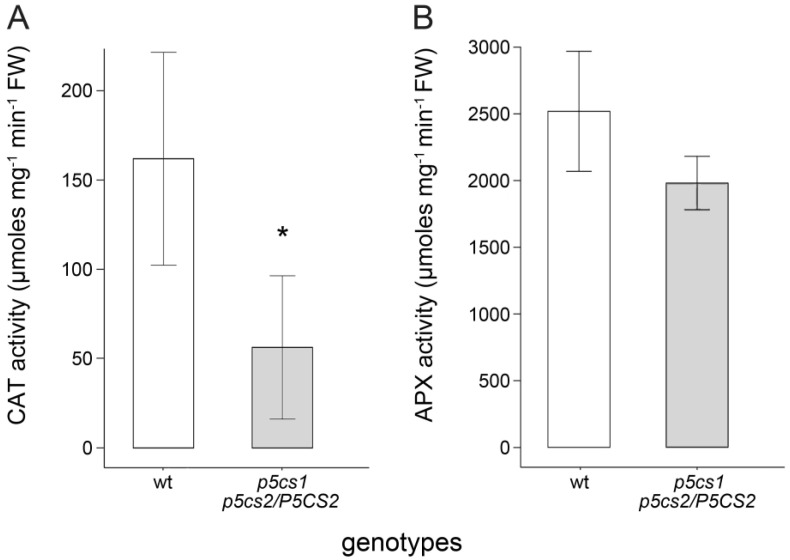
Enzymatic activity of catalase (CAT) and ascorbate peroxidase (APX) in wildtype and *p5cs1 p5cs2/P5CS2* roots. CAT (**A**) and APX (**B**) are considered the most effective scavengers of H_2_O_2_ in the plant cell. The values are the means of four independent experiments. Error bars indicate Standard Deviation (SD). The statistical significance (* *p* < 0.05) was calculated with a Welch test. The data represent the means ± SD of at least four independent experiments and three technical replicates per experiment.

Although the increased expression of many antioxidant genes does not correlate with the low proline levels, the enzymatic activity of these enzymes might do. Several authors reported that proline enhances the activity of various antioxidant enzymes [23,24,41,42]. To explore this possibility, we analyzed protein extracts from sesquimutant and wildtype roots for the enzymatic activity of catalase (CAT), and ascorbate peroxidase (APX), the two main H_2_O_2_-scavenging enzymes in plants [43]. We consistently found less CAT and APX activity in protein extracts from proline-deficient mutants, although only CAT activity was significantly lower (* *p* < 0.05) in *p5cs1 p5cs2/P5CS2* than in wildtypes (Figure 10). This finding is consistent with the hypothesis that proline metabolism can modulate H_2_O_2_ levels by enhancing the activity of antioxidant enzymes. Exogenous proline added to the enzymatic assays at different concentrations from 10 to 1000 µM produced no effects on enzymatic activities (not shown), indicating that proline has no direct effect on enzyme activity, at least at these concentrations.

## 3. Discussion

In this work, we investigated the relationships between proline and ROS to clarify the molecular mechanism underlying the effects of proline on root meristem. The possibility of such an interaction is suggested by early reports claiming a role for proline as a ROS scavenger [15] and by the works of Dunand et al. [13] and Tsukagoshi et al. [14] who showed that the ratio between superoxide anion and hydrogen peroxide affects root meristem growth in a hormone-independent manner, similarly to proline. Furthermore, changes in ROS distribution have been shown to modulate root meristem size by affecting the stability of the PLETHORA2 protein, a master regulator of root stem cells in *Arabidopsis* [44]. Consistently, by controlling ROS abundance and distribution, proline can potentially modulate any developmental process downstream of ROS signaling, including root growth. Indeed, by treating root meristems with visual markers for O_2_^•–^ and H_2_O_2_, we consistently found a different pattern of accumulation of these ROS in the root meristems of wildtype and *p5cs1 p5cs2/P5CS2* mutants. In addition, supplementation of exogenous proline strongly altered the ROS distribution in the *Arabidopsis* root meristem, confirming the effect of proline on ROS balance. Similar to the findings of Dunand et al. [13] and Tsukagoshi et al. [14], we found that proline-mediated root elongation is associated with increased superoxide and decreased hydrogen peroxide. Although we found most of the NBT staining localized in the root meristem, and the DAB staining shifted towards the elongation/differentiation region, we were unable to precisely confirm the ROS localization proposed by Dunand because of the low sensitivity of the staining methods.

The effects of proline on the root meristem turned out to be similar to those induced by H_2_O_2_. Indeed, both molecules act independently from plant hormones [9,14] and play a similar dual role by stimulating root elongation and cell cycle activity at low concentrations and becoming toxic at higher concentrations, although within different concentration ranges [45,46,47]. The stimulatory effect on root growth and cell cycle activity of either proline or H_2_O_2_ suggests a role in signaling. Indeed, both proline and H_2_O_2_ have been implicated in signal transduction in different physiological and biochemical processes in plants including seed germination [48], senescence [49], embryogenesis [10,11], root system development [50,51,52], root elongation [9,47] pollen development [53,54,55,56], control of stomatal aperture [57,58], and flowering time [59,60].

The signaling role of H_2_O_2_ is particularly well established. It is well documented, for example, that the *Arabidopsis* gene *OXI1*, encoding a serine/threonine kinase, is upregulated by a range of H_2_O_2_-generating stimuli, and its kinase activity is enhanced by H_2_O_2_ in vitro [61]. Upon ROS induction, OXI1 kinase activates the protein kinases MPK3 and MPK6 to participate in diverse downstream responses. MAPK cascades, involving MKK4/5 and MPK3/6, have been shown to respond to H_2_O_2_ to activate antioxidant activities [62]. We now know that H_2_O_2_ triggers an intracellular influx of calcium ions (Ca^2+^), which is transmitted in waves across cells, eventually leading to the activation of several downstream processes, such as pathogen resistance or stress tolerance [63]. Moreover, H_2_O_2_ regulates pollen tube elongation, root hair growth, and stomatal closure [64]. Recently, Wu et al. [65] identified HPCA1, a membrane-spanning leucine rich repeat (LRR) receptor kinase, as the first extracellular sensor of hydrogen peroxide in plants.

A signaling role for proline is less clear, although it has been hypothesized by several authors upon circumstantial evidence [59,66]. Moustafa et al. [67] found in *Arabidopsis* that both *MPK20* and ProDH exhibit a similar pattern of expression upon hypoosmotic stress, suggesting a link between proline metabolism and MAP kinases. Incidentally, a genomic study [68] revealed that in *Gossypium raimondii, MPK20* is strongly induced by H_2_O_2_, suggesting that the signaling effects of proline metabolism may be triggered by ROS signaling. Furthermore, Zarse et al. [69] report that in *Caenorhabditis elegans,* proline metabolism promotes insulin and IGF-1 signaling to generate a ROS signal to incite endogenous stress defense and extend life span. Incidentally, transgenic tobacco plants ectopically expressing *rolD*, a plant oncogene encoding a proline synthesis enzyme of bacterial origin [70,71,72], exhibited early flowering and extended life span.

As also reported by other authors [73,74,75], at high concentrations, proline becomes progressively toxic for plant cells, similarly to H_2_O_2_, but at higher dosages. The cause of proline toxicity is not fully understood but might be caused by the accumulation of pyrroline-5-carboxylate, an intermediate of proline catabolism shown to be highly toxic to plants [73,74,76], yeast [77,78], and animal cells [79]. An alternative explanation postulates that unbalanced proline catabolism, rather than P5C accumulation, may lead to cell toxicity. Proline catabolism is a rich source of energy, capable of producing 30 ATP equivalents per molecule [80], and it is well suited to sustain the needs of energy-demanding biological processes, such as root elongation, bolting, and pollen tube elongation. Some flying insects, such as butterflies and bees, use proline in the first and more expensive stage of the flight as a readily-available, highly-energetic boost [81]. During proline catabolism, however, ROS are also produced as a by-product of mitochondrial respiration. In the inner mitochondrial membrane, ProDH catalyzes the FAD-dependent oxidation of proline to P5C and transfers electrons directly to the electron transport chain, generating O_2_^•–^ and, in turn, H_2_O_2_ [3,82]. Once the H_2_O_2_ concentration exceeds the enzymatic and non-enzymatic scavenging potential of the plant cell, toxicity and cell death occur. Moreover, the evidence that *prodh* mutants, despite their lack of the first step of proline degradation, are hypersensitive to exogenous proline applications, as reported by [75,83] and experimentally confirmed in this work (not shown), may further suggest that P5C and O_2_^•–^ produced by proline degradation are not the only effectors of proline toxicity. What exactly causes cell toxicity at high proline concentrations and why *prodh* mutants are hypersensitive to exogenous proline remains to be understood.

Through genetic and pharmacological experiments, we clearly showed that the effect of proline on the root meristem depends on a complex interaction between proline and H_2_O_2_, which is epistatic over proline and represents the main effector of root modulation. Accordingly, by treating *p5cs1 p5cs2/P5CS2* roots with KI, a strong and effective inhibitor of H_2_O_2_ accumulation, we fully complemented the short-root phenotype of the proline-deficient mutant, strongly suggesting that the effect of proline deficiency on root meristem size is only indirect and mediated by H_2_O_2_. We reached similar conclusions by crossing *p5cs1 p5cs2/P5CS2* with *upb1*, a mutant allele of *UPBEAT* [14] with increased peroxidase activity and low levels of H_2_O_2_. The root meristems of the resulting quasi-triple mutants grew roots of intermediate size between the parental lines, suggesting that the low amount of H_2_O_2_ of the *upb1* mutants counteract the high levels of H_2_O_2_ of the *p5cs1 p5cs2/P5CS2* mutants, leading to partial complementation of the root elongation defect of proline mutants.

The role of O_2_^•–^ in proline metabolism, however, is less well-defined in this work, although is very clear that proline has a profound impact on O_2_^•–^ distribution. According to Dunand et al. [13], and Tsukagoshi et al. [14], O_2_^•–^ is necessary for root elongation, and one may expect a short-root phenotype in mutants lacking ProDH activity. In reality, both *prodh1 prodh2* and *prodh1 prodh2 p5cs1 P5CS2/p5cs2* have large root meristems that contain low levels of H_2_O_2_. By contrast, the low level of H_2_O_2_ found in *prodh1 prodh2* and *p5cs1 p5cs2/P5CS2 prodh1 prodh2* is consistent with their lack of proline catabolism. Importantly, the low level of proline detected in *p5cs1 p5cs2/P5CS2 prodh1 prodh2* is in contrast with the low levels of H_2_O_2_ of this mutant, strongly suggesting that proline catabolism and not proline itself is the cause of the H_2_O_2_ reduction, a question long debated among researchers [1]. It must be noted, however, that the actual levels of proline accumulation in root meristems may not reflect the amount of proline measured in seedlings because of specific transport of proline to root tips [84], and further work to measure proline levels within root meristems is clearly required.

**Figure 11:**
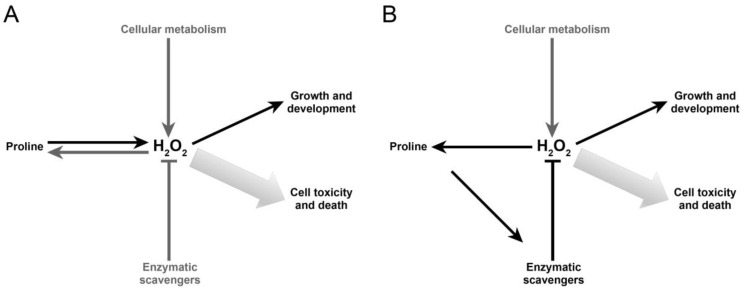
Possible models of interactions between proline and ROS. Proline catabolism stimulates the accumulation of H_2_O_2_ production, which, in turn, can induce proline synthesis (**A**). Proline seems not able to scavenge directly H_2_O_2_ but might indirectly control its accumulation by enhancing the activities of key antioxidant enzymes (**B**).

Based on evidence reported in this work, proline seems to exert its effects on the root meristem by modulating the levels of H_2_O_2_, which stimulates root elongation at low concentrations, and cell death at high concentrations. We are aware that this is a very simplistic model, as cellular metabolism continuously generates H_2_O_2_ from different sources, while several enzymatic and non-enzymatic activities counteract H_2_O_2_ accumulation. In addition, H_2_O_2_ itself has been reported to induce proline synthesis [27], adding complexity to this regulatory network. It is tempting to speculate that proline may behave as an H_2_O_2_ integrator in response to developmental and environmental stimuli and coordination with other enzymatic and non-enzymatic activities (Figure 11A).

More difficult to understand is how proline can affect the levels of H_2_O_2_ in the root meristem. The analysis of expression of the main genes coding for ROS scavengers produced unexpected results since we found most of these genes upregulated in the *p5cs1 p5cs2/P5CS2* mutant. It is difficult to reconcile this result with the reduced capacity of the mutant to scavenge H_2_O_2_. The more likely explanation is that the high level of expression of the genes coding for antioxidant enzymes is caused by the high level of H_2_O_2_ that accumulates in the mutant roots. On the contrary, the enzymatic activity of CAT and APX, two of the main H_2_O_2_-scavenging enzymes of the plant cell, appeared reduced in *p5cs1 p5cs2/P5CS2* (although only CAT activity was significantly reduced with a *p* < 0.05), suggesting that proline controls H_2_O_2_ accumulation by enhancing the activity of some antioxidant enzymes—at least CAT and APX. This evidence is in line with previous reports [22,23,24] and refines the original model by including a regulatory loop among proline, H_2_O_2_, and antioxidant enzymes that well fits a hypothetical role of proline as a regulator of H_2_O_2_ homeostasis (Figure 11B).

At present, we still do not know how proline enhances the activity of these enzymes and can only speculate on a possible model. Since a direct interaction between proline and H_2_O_2_ seems unlikely [1,85]; this work), and the correlation between proline and growth stimulation is not always held, the scavenging properties of proline may lay in its metabolism, perhaps by regulating the ratio between NAD(P)^+^ and NAD(P)H. During proline synthesis, glutamate and P5C reduction are coupled to NADPH oxidation to regenerate NADP^+^ and fuel the activity of glucose-6-phosphate dehydrogenase—the rate-limiting enzyme of the pentose phosphate pathway (PPP) in plants. PPP is the main source of cytosolic NADPH which is needed to regenerate oxidized glutathione and feed the ascorbate-glutathione pathway, one of the most effective scavenging systems in the cell. Accordingly, the alteration of the NADP^+^/NADPH ratio modulated by proline synthesis could potentially affect the antioxidant potential of the plant cell.

Furthermore, proline-induced PPP can potentially stimulate shikimate and phenylpropanoid pathways, leading to the production of anti-oxidant polyphenol molecules, which may further improve the proline-induced scavenging power. Last but not least, proline itself can potentially improve the activity of the anti-oxidant enzymes thanks to its kosmotropic properties [1]. A delicate balance between ROS production and scavenging, involving proline, ROS, and perhaps hormonal pathways, must be maintained. Once the metabolic and enzymatic buffer system is saturated, excessive proline leads to H_2_O_2_ overproduction, inhibition of cell growth, and, ultimately, cell death, possibly through the macroautophagy/autophagy pathway [86].

Overall, we showed that the effect of proline on the root meristem size of *Arabidopsis* clearly exceeds its function in protein synthesis and seems to be mediated by a modulatory role in ROS homeostasis. Proline modulation of ROS homeostasis seems to involve proline metabolism rather than proline itself and likely triggers a signaling cascade, eventually affecting plant development.

## 4. Materials and Methods

### 4.1. Plant Growth Conditions and Genetic Crosses

Wildtype and mutant *Arabidopsis thaliana* (L) Heynh., ecotype Columbia-0 (Col-0), were grown in a growth chamber at 24/21 °C with a light intensity of 300 μE m^−2^ s^−1^under 16 h light and 8 h dark per day. Seeds were surface-sterilized, stratified for three days at 4 °C, and germinated on 1⁄2xMurashige and Skoog (MS) plates [87]. *Arabidopsis* lines homozygous for *p5cs1* and heterozygous for *p5cs2 (p5cs1 p5cs2/P5CS2*) have been characterized and described in [11,88]. Heterozygous *p5cs1 p5cs2/P5CS2* mutants were selected on plates containing 4 μg ml^−1^ sulfadiazine and the presence of the *p5cs2* mutant allele was occasionally confirmed by PCR analysis of random samples using either primers for the P5CS2:T-DNA junction or the sulfadiazine resistance gene. In preliminary experiments, we confirmed that the presence of sulfadiazine had no influence on root growth or the size of the root meristem of *p5cs1 p5cs2/P5CS2* mutants. Homozygous mutants *prodh1-1 prodh2-1* (referred to as *prodh1 prodh2*)*,* have been described in [34]. Quasi-quadruple mutants *prodh1 prodh2 p5cs1 p5cs2/P5CS2* were generated by manually cross-pollinating *p5cs1 p5cs2/P5CS2* flowers with *prodh1 prodh2* pollen and the selection of sulfadiazine-resistant plants homozygous for *prodh1-1*, *prodh2-1* and *p5cs1-4* in the F2 and F3 generation. *Upb1* mutants were kindly provided by Philip Benfey, Duke University. Quasi-triple mutants *upb1 p5cs1 p5cs2/P5CS2* were generated by manually cross-pollinating *p5cs1 p5cs2/P5CS2* flowers with *upb1* pollen. In all genetic crosses using *p5cs1 p5cs2/P5CS2*, the mutant used was a female because of the male sterility of the *p5cs1 p5cs2* pollen grains [53].

### 4.2. Analysis of the Root Meristem

To analyze root meristem size, seedlings from *p5cs1 p5cs2/P5CS2* and wildtype plantlets were grown on vertical plates and analyzed at 3, 5, or 7 days after germination (DAG). Although we obtained similar results in all the time points analyzed, the best staining results were produced at 5 DAG, and, accordingly, all data and pictures of NBT- or DAB-stained samples were taken at 5 DAG. In contrast, all data and pictures of meristem counts were taken at 7 DAG when the root meristem reached its final dimension. To measure root meristem size, we counted the number of the cortex meristem cells spanning from the quiescent center to the first elongated cell using an Axioskop 2 light microscope equipped with Nomarski optics (Carl Zeiss Microimaging GmbH, Jena, Germany). Digital images were acquired with a Jenoptik ProgResW C3 digital camera (Jenoptik, Jena, Germany). Prior to observations, roots were treated with a chloral hydrate solution until the stem cell’s niche was clearly visible. NBT and DAB assays were performed according to Kumar et al. [89] with minor modifications. The intensity of NBT and DAB staining in the root meristem was determined by scanning digital pictures with ImageJ [28] software (http://rsb.info.nih.gov/ij accessed on 15 June 2020) and selecting, with the polygon tool, the region roughly spanning from the staminal niche to the elongation/differentiation zone. Proline, KI, and H_2_O_2_ supplementations were made by adding the appropriate concentrations to Petri plates and letting plantlets germinate and grow as specified in the text. All the analyses have been repeated at least three times in independent experiments. Statistical analyses were performed with R version 3.6.3 (R core team, 2019). We used either one-way or two-way ANOVA whenever the required normality and homoscedasticity assumptions were satisfied. In other cases, we used the paired parametric Welch t-test. GUS staining was carried out by infiltrating roots under a vacuum for 1 h and incubating them overnight in an X-Gluc solution at 37 °C [90].

### 4.3. Proline Analysis

Proline content was measured according to [37], using L-proline as a standard. The absorbance was read at 520 nm with a NanoDrop 2000 spectrophotometer (Thermo Fisher Scientific, Waltham, MA, USA). Proline content was determined relative to the fresh weight and normalized to the value of wildtype seedlings. Every measurement represents the average from more than 100 14-day-old seedlings coming from at least three independent experiments. Since the samples did not pass the normality test, we estimated statistical significance with a non-parametric Kruskal–Wallis test, followed by a pairwise Wilcox test.

### 4.4. Molecular Techniques

Molecular techniques were performed according to standard protocols. Total RNA for RT-qPCR was extracted from roots using NucleoSpin RNA Plant (Macherey-Nachel, Hoerdt, France) according to the manufacturer’s instructions. RNA quality (A_260_/A_280_ ratio >2) and quantity was assessed with a NanoDrop 1000 (Thermo Fisher Scientific, Milan, Italy). Reverse transcription was performed from 1 μg of total RNA using the QuantiTect Reverse Transcription kit (Qiagen, Hilden, Germany) as recommended by the manufacturer. For genomic PCR, *Arabidopsis* DNA was extracted with a modified CTAB method, according to Stewart et al. [91]. Primers and PCR conditions used for *p5cs1* and *p5cs2* were already described [11]. Real-time RT-qPCR analyses were carried out with a Rotor-Gene Q (Qiagen, Hilden, Germany). Amplifications were monitored using the SYBR Green fluorescent stain. The presence of a single PCR product was verified by dissociation analysis in all amplifications. All the primers used for the RT-qPCR analysis were designed with Primer-BLAST (https://www.ncbi.nlm.nih.gov/tools/primer-blast/ accessed on 10 March 2021) and reported in Table 1. Primer efficiency was determined for each pair of primers by amplifying serial dilutions of target genes and plotting the resulting Cq against the log-transformed value of the dilution. The comparative threshold cycle (ΔΔCq) method was used to calculate the relative amount of gene expression and normalized using the Cq values of a normalizer gene. Because we were interested in root-meristem expression, we synthesized cDNA from the apical portion of either sesquimutant or wildtype roots and normalized the expression of target genes to the root-meristem specific gene RCH1 [38], as described in [9].

### 4.5. Enzymatic Assays

For the catalase assay, about 300 mg (FW) of 7-day-old seedlings were homogenized with a glass potter in 1.5 mL of 50 mM potassium phosphate buffer (pH 7.0) The homogenate was centrifuged for 15 min at 4 °C at 20,000× *g*, and the resultant supernatant was precipitated with 55% ammonium sulfate, redissolved in 100 µL potassium phosphate buffer, and extensively dialyzed in the same buffer. The catalase assay was carried out according to [93] by mixing in a 1 mL quartz cuvette 50 mM of potassium phosphate buffer (pH 7.0), 30 μL of enzyme extract, and 60 mM of hydrogen peroxide. To measure CAT activity, we followed the degradation of H_2_O_2_ by measuring, with a Hitachi U-2000 spectrophotometer, the reduction in its absorbance at 240 nm over 3 min. Catalase activity was calculated according to [93] using the extinction coefficient for H_2_O_2_ (ε = 36 M^−1^ cm^−1^) and expressing the activity as µmol mg^−1^ min^−1^. For the APX assay, about 100 mg (FW) of 7-day-old seedlings were homogenized with a glass potter in 1.5 mL of 50 mM potassium phosphate buffer (pH 7.0), containing 5 mM ascorbate peroxidase. The homogenate was centrifuged at 20,000× *g* for 15 min at 4°C, and the resultant raw supernatant was used for enzymatic assays. The APX assay was carried out in a 1 mL quartz cuvette containing 50 mM sodium phosphate buffer (pH 7.0), 0.2 mM EDTA, and 5 mM ascorbate peroxidase. The enzyme activity was assayed by measuring, over 3 min, the decrease in absorbance of ascorbate at 290 nm. APX activity was calculated, according to [94], using the extinction coefficient for ascorbate (ε = 2.8 mM^−1^ cm^−1^) and expressing the activity as µmoles cm^−1^mg^−1^min^−1^. Protein concentrations in plant extracts were determined with a Bradford assay [95] using bovine serum albumin as a standard.

## 5. Conclusions

In conclusion, we showed that proline metabolism, but not proline itself, modulates root meristem size by controlling the activities of ROS-scavenging enzymes and, ultimately, H_2_O_2_ accumulation. The control of proline on ROS distribution is likely exerted by the fine-tuning of the cellular redox balance and may represent a general mechanism to explain the multiple effects of proline in stress and development. Several questions, though, remain unanswered. It is still unclear, for example, how proline affects the activity of antioxidant enzymes, how the plant manages the delicate trade-off between proline synthesis and catabolism, and which ROS-induced genes are responsible for the modulation of root meristem size in response to intracellular proline levels. Future experiments are clearly required to find an answer to these long-awaited biological questions.

## Figures and Tables

**Table 1:** List of the primers used for RT-qPCR.

Primer Name	Sequence	Gene
RCH1_for	GGCGTGTTGGCGGTTATACG	At5g48940
RCH1_rev	ATCCCGGAGCAACCTTTCCC	
CYCB1;1_for	TGGTAGCTGCTTCTGCAATC	At4g37490
CYCB1;1_rev	AGCTTTGCACAGTCCATGAG	
CAT1_for	TCTCCCACCACCCAGAGAGT	At1g20630
CAT1_rev	AGCTTCCTCATCCGACAGGC	
APX1_for	CGTCCATTTTAAAGCCGTGCG	At1g07890
APX1_rev	CGAGTGGCTGGCACGAGTAA	
DHAR1_for	CCCACTGGTGGGTGGAGAAT	At3g24170
DHAR1_rev	CGGACAGTCGCCGAGATGAT	
CSD1_for	AGCAGTGAGGGTGTTACGGG	At1g08830
CSD1_rev	GGGGCACCGTGTGTTTTACC	
MSD1_for	TTCAACGGCGGAGGTCATGT	At3g10920
MSD1_rev	AGCCACCATCCTGAGCCTTG	
Per_39_for	AAGCTTGCTCCTCCGAATCT	At4g11290
Per_39_rev	GTCGGTCCACCAATAGCAAC	
Per_40_for	CTTGGCCTTTCACAAACCGA	At4g16270;
Per_40_rev	TGGTTGTCCAGTTTGCAGTG	
Per_57_for	AAGCTTGCTCCTCCGAATCT	At5g17820
Per_57_rev	GTCGGTCCACCAATAGCAAC	

Note: The root meristem-specific gene *RCH1* (ROOT *CLAVATA HOMOLOG1*) is also known as *RGFR2* (*RGF1 INSENSITIVE 2*) [92].

## Data Availability

Not applicable.
